# Effect of rapid antiretroviral therapy initiation on loss to follow-up, virologic failure, and immunologic failure among people living with HIV under the treat-all policy in China: a retrospective cohort study (2016–2023)

**DOI:** 10.3389/fmed.2026.1882194

**Published:** 2026-08-11

**Authors:** Shuanglian Ao, Yan Zheng, Liuying Yang, Zongyu Nie, Peng Li, Hai Long

**Affiliations:** 1 School of Public Health, Guizhou University of Traditional Chinese Medicine, Guiyang, China; 2 Guiyang Public Health Clinical Center, Guiyang, China; 3 School of Geography and Environmental Science/Karst Research Institute, Guizhou Normal University, Guiyang, China

**Keywords:** antiretroviral therapy, China, HIV, immunologic failure, loss to follow-up, rapid initiation, virologic failure

## Abstract

**Introduction:**

Since 2016, China has fully implemented the Treat-All policy, which eliminated the CD4 cell count threshold and ensures timely antiretroviral therapy (ART) for all people living with HIV (PLWH). This study aimed to evaluate the impact of rapid ART initiation ( ≤ 7 days post-HIV diagnosis) versus delayed initiation on loss to follow-up (LTFU), virologic failure, and immunologic failure among PLWH.

**Methods:**

This study enrolled newly diagnosed, ART-naive adult PLWH in Guizhou Province, China, between 2016 and 2023. Kaplan–Meier analysis was used to examine LTFU across groups. Multivariable Cox regression was employed to evaluate the association between rapid ART initiation and LTFU, and logistic regression with multiple imputation was used to assess 12-month virologic and immunologic outcomes.

**Results:**

Among 8163 participants, 2604 (31.9%) initiated ART within 7 days of diagnosis. The rapid ART initiation rate increased from 26.5% in 2016 to 47.0% in 2023. The LTFU rate was comparable between rapid and delayed (≥30 days) initiators (31.2% vs. 32.5%, *P* = 0.120). Compared with deferred ART, immediate rapid initiation was associated with significantly higher odds of virologic failure (aOR: 1.406; 95% CI: 1.020–1.940; *P* = 0.038) and immunologic failure (aOR: 1.540; 95% CI: 1.221–1.941; *P* < 0.001).

**Discussion:**

Real-world data indicate that rapid ART initiation does not reduce LTFU risk, but significantly elevates the dual risks of virologic and suboptimal immunologic failure. To achieve clinical benefits when scaling up rapid ART in resource-limited settings, initiation pathways must be paired with individualized, intensified pre-ART counselling and comprehensive adherence support.

## 1 Introduction

Over the past 2 decades, antiretroviral therapy (ART) has served as a cornerstone strategy worldwide to combat the human immunodeficiency virus (HIV)/acquired immunodeficiency syndrome (AIDS) pandemic. It can effectively suppress viral replication, promote immune reconstitution, prolong the survival of people living with HIV/AIDS, and reduce the risk of subsequent viral transmission (1, 2). The World Health Organization (WHO) 2015 treatment guidelines recommended that all PLWH receive ART, regardless of CD4 cell count (3). Nevertheless, new HIV infections continue to occur, particularly among high-risk populations such as men who have sex with men (MSM) and in key geographic areas (4–6). Early initiation of ART can effectively suppress viral load, reduce HIV transmission risk, and thereby decrease new HIV incidence, Ideally, ART should be initiated immediately upon HIV diagnosis. In 2017, WHO updated guidelines based on clinical findings of same-day and rapid ART, which recommended that PLWH without opportunistic infection symptoms initiate ART on the day of HIV diagnosis or within 7 days (7–9).

In 2003, China established the National Free Antiretroviral Treatment Program (NFATP), which significantly improved the accessibility of ART among people with HIV (PLWH), effectively reduced AIDS-related mortality in this population, and led to a sustained and rapid increase in the number of PLWH receiving government-funded free ART (10, 11). Initially, the NFATP recommended ART initiation for PLWH, but restricted treatment provision to those with < 200 cells/μL CD4^+^ T cell count or WHO clinical stage 4 (AIDS stage). From 2008 to 2014, the primary eligibility criterion for NFATP was a CD4^+^ T cell count ≤ 350 cells/μL, which was further expanded to ≤ 500 cells/μL in 2015 (12). In accordance with WHO guidelines, China has implemented the “Treat-All” policy since 2016, removing CD4 count restrictions and recommending early ART initiation for all PLWH upon diagnosis (13, 14). The updated Chinese Guidelines for HIV/AIDS Diagnosis and Treatment (2021 edition) recommend rapid ART initiation or same-day ART for eligible PLWH (15). By the end of 2022, 1.223 million reported PLWH were alive in China, among whom 92.8% were receiving ART (16, 17).

Between 2011 and 2015, a large-scale cohort study conducted in China showed that immediate ART initiation (within 30 days of HIV diagnosis) was associated with lower all-cause mortality, LTFU, and virologic failure among all PLWH (18, 19). Most current studies of rapid ART initiation predominantly focus on Africa, whereas real-world evidence from southwestern China under the national Treat-All policy is still limited. Therefore, this study aimed to evaluate the associations between rapid ART initiation and LTFU, virologic failure, and immunologic failure in a large cohort of PLWH in Guizhou, China.

## 2 Methods

### 2.1 Study setting and design

This retrospective observational study included newly diagnosed HIV-positive adults identified between June 2016 and June 2023 in Guizhou Province, China, a provincial region with a moderate HIV prevalence (PLWH >10,000). Exclusion criteria included individuals who (1) HIV diagnosis before the implementation of the national universal treatment policy; (2) aged < 18 years; (3) had prior ART experience; (4) < 12 months between enrollment and most recent visit; (5) never initiated ART; and (6) transferred out.

Data were obtained from the National HIV/AIDS Comprehensive Response Information Management System (CRIMS) of the Chinese Center for Disease Control and Prevention (CDC) (20) and hospital electronic medical records (EMR). Data collected included sex, age, marital status, HIV diagnosis dates, transmission routes, viral load, baseline CD4 count, HIV treatment enrollment, ART prescription date, initial ART regimen, clinical visits, transfer to other cities, and mortality data. The initial ART regimen was defined as antiretroviral drugs prescribed at the time of ART initiation. The initial ART regimen was defined as antiretroviral drugs prescribed at the time of ART initiation. In addition, baseline CD4 count was assessed using test data obtained within 3 months prior to ART initiation.

### 2.2 Outcome measures

Participants were categorized based on the time interval from HIV diagnosis to ART initiation: ≤ 7 days, 8–29 days, and ≥30 days. In line with WHO guidelines and operational research definitions, rapid ART initiation was defined as ART initiation ≤ 7 days postdiagnosis, and delayed ART initiation as initiation >7 days after diagnosis (21). The primary treatment outcome was 12-month LTFU, defined as no documented contact with healthcare facilities >90 days after the last drug dispensing or clinical visit within 12 months post-enrollment, among participants who had not died or been transferred out (22–24). The secondary treatment outcome was 12-month virologic failure (defined as a viral load ≥200 copies/mL at the first annual test post-ART initiation). Immunologic failure at the 12-month follow-up was defined as the tertiary study endpoint, with diagnostic criteria formulated according to the National Free Antiretroviral Treatment Guidelines for HIV/AIDS (4th Edition) (25). This guideline serves as the unified official clinical reference for HIV care facilities nationwide, including the regional healthcare system that generated our study cohort. Adopting this localized standard helped align our findings with routine clinical practice and objectively reflects the real-world implementation of national HIV public health policies. Specifically, Immunologic failure was identified at least 6 months after ART initiation, regardless of virologic suppression status, if either criterion was satisfied: (1) CD4^+^ T-cell count dropped to or below the pre-treatment baseline value; (2) sustained CD4^+^ T-cell counts less than 100 cells/μL.

### 2.3 Statistical analysis

All analyses were performed using SPSS version 26.0 (SPSS, Chicago, IL). Continuous variables were presented as medians and interquartile ranges (IQRs), while categorical variables were expressed as frequencies and percentages. Differences in medians of continuous variables were evaluated using the Kruskal–Wallis tests, whereas differences in categorical variables were assessed with the chi-square test. Time-to-event analysis was performed, with follow-up duration measured in days from ART initiation to the final follow-up within a 12-month follow-up period. The Kaplan–Meier method was used to estimate LTFU rates and compare differences between ART initiation timing groups. Initially, a multivariable Cox regression model was uesd to identify factors associated with 12-month LTFU after enrollment. We evaluated the proportional hazards (PH) assumption using covariate-specific Schoenfeld residual tests alongside log-log survival curves. For all predictors exhibiting significant time-dependent hazard effects, we fitted an extended Cox model with covariate-follow-up time interaction terms to correct violations of the PH assumption. The discriminative ability of each regression model was measured by Harrell's C-index. Global model fit was summarized with the −2 log-likelihood statistic, Akaike Information Criterion (AIC), and Bayesian Information Criterion (BIC). To mitigate potential mediation and overadjustment bias associated with the initial ART regimen, this covariate was omitted from the primary analytic model. Its potential association with LTFU was evaluated in a separate hierarchically adjusted sensitivity Cox model. Effect sizes for LTFU outcomes were presented as adjusted hazard ratio (aHR) and time-varying aHR generated from interaction terms, together with corresponding 95% confidence intervals (CIs).

In addition, we fitted univariable and multivariable logistic regression models to identify independent correlates of virologic failure and immunologic non-response. The discriminative performance of each adjusted multivariable model was evaluated by the area under the receiver operating characteristic curve (AUC), and model calibration was validated via the Hosmer–Lemeshow goodness-of-fit test. Missing laboratory follow-up measurements were managed using multiple imputation by chained equations (MICE). Sensitivity analyses incorporating diverse missing-data and sample-handling strategies were further conducted to verify the robustness of the logistic regression results. Effect sizes were quantified as crude odds ratio (OR), adjusted odds ratio (aOR), and 95% confidence interval (95% CIs). All *P* values were two-sided, and *P* < 0.05 was considered statistically significant.

### 2.4 Handling of missing date

We addressed missing values in two distinct categories: baseline covariates and longitudinal follow-up endpoints.

#### 2.4.1 Baseline missing data

Among the original screening cohort of 57,892 patients, baseline biomarker missingness was extensive; 34.6% of participants had no recorded CD4^+^ T-cell counts, and 77.4% lacked baseline HIV viral load measurements. Such missingness mainly arose from incomplete baseline clinical workups—including early death, rapid LTFU, or inter-facility care transfer—circumstances that strongly suggest a Missing Not At Random (MNAR) mechanism. Given this non-ignorable missing structure and the high missing proportion, multiple imputation was deemed statistically inappropriate. Accordingly, we excluded patients with incomplete baseline data from the primary analytical cohort and adopted a complete-case strategy for all baseline variables.

#### 2.4.2 Follow-up missing endpoints

Among the 8,163 patients retained in the final analytical cohort, missing values existed for the 12-month virologic and immunologic follow-up endpoints. For the primary analyses, we imputed missing laboratory outcome data using Multiple Imputation by Chained Equations (MICE). In addition, we conducted extensive sensitivity analyses using a range of alternative missing-data handling strategies to evaluate the robustness of our findings under varying missingness assumptions. Full technical details regarding these model specifications and analytical frameworks are elaborated in Section 2.5 of the main manuscript.

### 2.5 Model specifications for sensitivity analysis

We conducted stratified sensitivity analyses using Cox proportional hazards and logistic regression models to evaluate the robustness of our findings under alternative covariate specifications and missing-data handling strategies. Four Cox models were defined based on two modifying factors: adjustment for the ART regimen and the inclusion of time-by-covariate interactions. Similarly, logistic regression analyses incorporated four distinct missing-data handling approaches (complete-case analysis, MICE, missing 12-month outcomes coded as virologic/immunologic failure, and MICE with retained LTFU). Each approach was stratified into unadjusted and ART-adjusted subsets, yielding eight model specifications. For both the Cox and logistic regression analyses, Model 3 served as the primary analytic model. Complete model details and all regression results are available in Supplementary Tables S3, S4.

### 2.6 Ethics approval

This retrospective study was approved by the Ethics Committee of Guiyang Public Health Clinical Center (Approval No.: (2025) KY-073, Approval Date: December 24, 2025). All procedures were conducted in accordance with the Declaration of Helsinki. Written informed consent was obtained from all participants for the use of their de-identified clinical records in this research, in accordance with the approved study protocol.

## 3 Results

### 3.1 Characteristics of the participants

**Figure 1:**
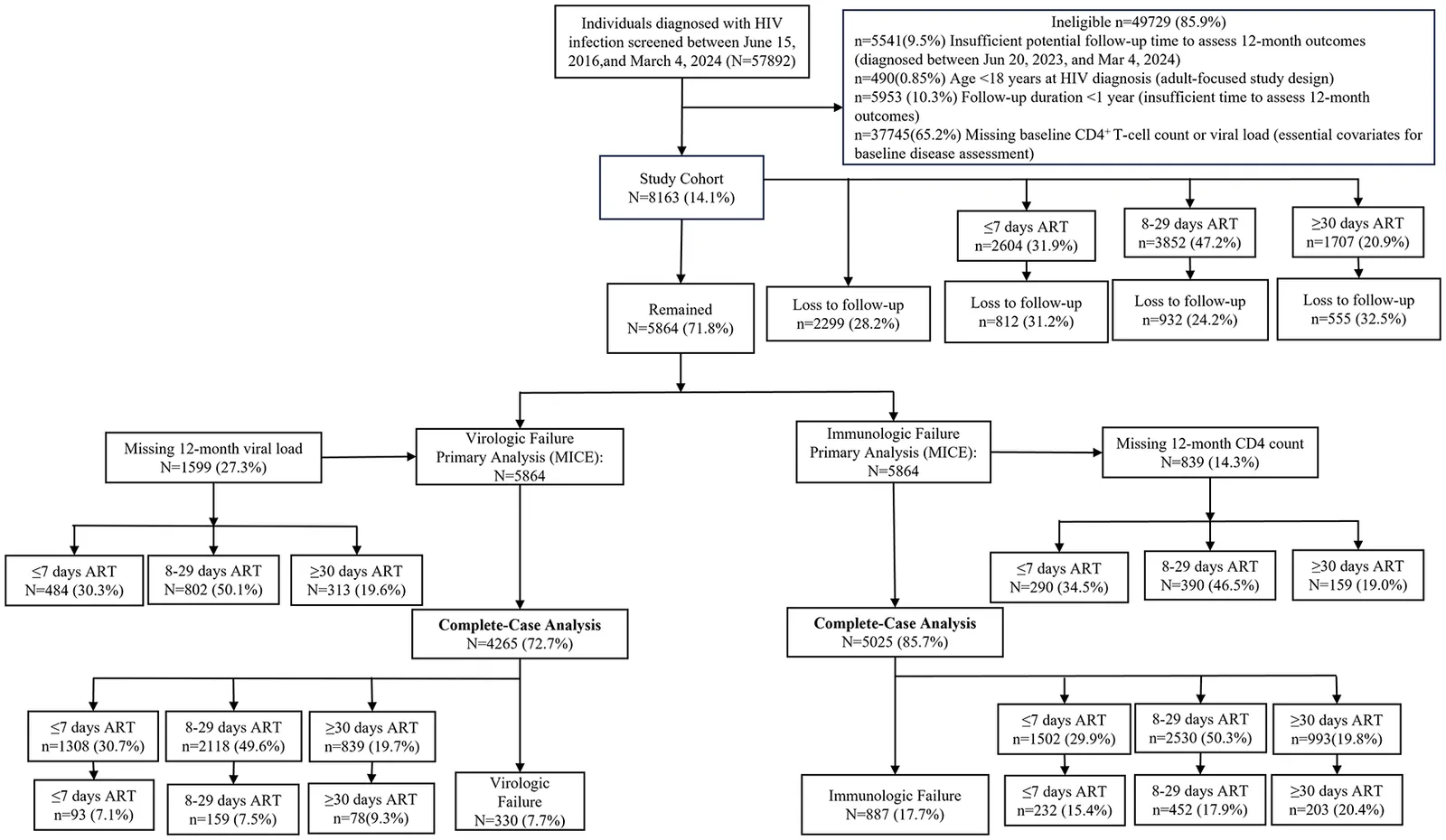
Flow diagram depicting study design and cohort assembly. All newly diagnosed persons with HIV who started ART between June 2016 and June 2023 were screened. Treatment outcomes included loss to follow-up, virologic failure (HIV RNA ≥ 200 copies/mL), and immunologic failure at 12 months after enrollment. ART, antiretroviral therapy; HIV, human immunodeficiency virus.

Among the initial screening cohort of 57,892 patients, 20,048 (34.6%) had missing baseline CD4^+^ T-cell counts, and 44,814 (77.4%) had no recorded baseline HIV viral load values. Patients missing these core laboratory biomarkers or failing to satisfy other eligibility criteria were excluded, leaving a final analytical sample of 8,163 participants (14.1%). The full patient selection flowchart and detailed exclusion rationales are presented in Figure 1. Compared with excluded individuals, participants retained for analysis were younger and had larger proportions of males, men who have sex with men (MSM), and patients prescribed INSTI-containing regimens. A complete baseline characteristic comparison between the two groups is available in Supplementary Table S1.

Table 1 shows the detailed baseline characteristics of the participants. In total, 2,604 individuals (31.9%), 3,852 individuals (47.2%), and 1,707 individuals (20.9%) initiated ART within ≤ 7 days, 8–29 days, and ≥30 days after diagnosis, respectively. The median times from HIV diagnosis to ART initiation for the three groups were 3 days (IQR: 1–6), 14 days (IQR: 10–20), and 65 days (IQR: 41–184), respectively (Table 1). A total of 70.3% of participants were male (5,741/8,163). Males accounted for 66.6% of rapid ART initiators. The median age at ART initiation was 47 years (IQR: 32–58), and 44.4% of participants were aged ≥50 years. In addition, participants aged ≥50 years accounted for 49.0% of those initiating ART within ≤ 7 days after diagnosis, followed by the 30–49 years group (35.6%). Over half of the participants (52.8%) initiated ART between 2021 and 2023. Among rapid ART initiators, 65.8% initiated treatment in this period, which was 1.37 times the proportion in the ≥30-day group (48.0%).

**Table 1:** Demographic details and other characteristics of participants.

Variables	Total (*n* = 8,163)	≤ 7 days ART (*n* = 2,604)	8–29 days ART (*n* = 3,852)	≥30 days ART (*n* = 1,707)	Statistic	*p*
Time from diagnosis to ART initiation (days)						
Median (IQR)	13 [6, 26]	3 [1, 6]	14 [10, 20]	65 [41, 184]	6,971.999	< 0.001
Sex, *n* (%)						
Female	2,422 (29.7)	871 (33.4)	1,106 (28.7)	445 (26.1)	30.116	< 0.001
Male	5,741 (70.3)	1,733 (66.6)	2,746 (71.3)	1,262 (73.9)		
Age, at enrollment (years), *n* (%)						
Median (IQR)	47 [32, 58]	49 [35, 60]	46 [32, 58]	44 [30, 57]	52.879	< 0.001
18–29 years	1,603 (19.6)	403 (15.5)	795 (20.6)	405 (23.7)	59.397	< 0.001
30–49 years	2,933 (35.9)	926 (35.5)	1,394 (36.2)	613 (35.9)		
≥50 years	3,627 (44.4)	1,275 (49.0)	1,663 (43.2)	689 (40.4)		
Enrollment period, *n* (%)						
2016–2020	3,850 (47.2)	890 (34.2)	2,073 (53.8)	887 (52.0)	260.389	< 0.001
2021–2023	4,313 (52.8)	1,714 (65.8)	1,779 (46.2)	820 (48.0)		
Enrollment CD4, (cells/μL), *n* (%)						
Median (IQR)	229 [119, 348]	237 [135, 354]	227 [112, 345]	219 [108, 347]	15.796	< 0.001
< 200	3,488 (42.7)	1,046 (40.2)	1,659 (43.1)	783 (45.9)	19.152	0.001
200–499	3,926 (48.1)	1,332 (51.2)	1,835 (47.6)	759 (44.5)		
≥500	749 (9.2)	226 (8.7)	358 (9.3)	165 (9.7)		
Baseline viral load, (copies/μL), *n* (%)						
Median (IQR)	60,700 [14,900, 222,000]	58,950 [14,301, 214,000]	64,950 [17,200, 245,000]	55,000 [11800, 199,000]	21.154	< 0.001
< 1,000	473 (5.8)	142 (5.5)	166 (4.3)	165 (9.7)	66.248	< 0.001
1,000–100,000	4,442 (54.4)	1,451 (55.7)	2,091 (54.3)	900 (52.7)		
>100,000	3,248 (39.8)	1,011 (38.8)	1,595 (41.4)	642 (37.6)		
Transmission route, *n* (%)						
Heterosexual	6,403 (78.4)	2,160 (82.9)	2,970 (77.1)	1,273 (74.6)	82.763	< 0.001
Homosexual	1,431 (17.5)	335 (12.9)	745 (19.3)	351 (20.6)		
Injection drug use	39 (0.5)	9 (0.3)	10 (0.3)	20 (1.2)		
Unknown	290 (3.6)	100 (3.8)	127 (3.3)	63 (3.7)		
Marital status, *n* (%)						
Single	2,132 (26.1)	550 (21.1)	1,046 (27.2)	536(31.4)	74.490	< 0.001
Married/cohabiting	4,149 (50.8)	1,399 (53.7)	1,990 (51.7)	760 (44.5)		
Divorced/widowed	1,882 (23.1)	655 (25.2)	816 (21.2)	411 (24.1)		
Initial ART regimen, *n* (%)						
NNRTI-based	5,962 (73.0)	1,953 (75.0)	2,747 (71.3)	1,262 (73.9)	45.582	< 0.001
PI-based	254 (3.1)	88 (3.4)	98 (2.5)	68 (4.0)		
INSTI-based	1,562 (19.1)	425 (16.3)	844 (21.9)	293 (17.2)		
Other	385 (4.7)	138 (5.3)	163 (4.2)	84 (4.9)		

ART, antiretroviral therapy; IQR, interquartile ranges; NNRTI, non-nucleoside reverse transcriptase inhibitor, including EFV, NVP, ETV/N; PI, protease inhibitor, including LPV/r, ATV/r, DRV/r; INSTI, integrase inhibitor, including DTG, RAL, EVG/c, BIC; Other: all initial ART regimens not classified as NNRTI, PI, or INSTI.

Baseline median CD4 cell counts and viral loads exhibited modest intergroup differences. The rapid ART group had the highest median CD4 count (237 cells/μL), whereas the ≥30-day group showed the lowest value (219 cells/μL). Median baseline viral load peaked among participants in the 8–29-day group and reached the minimum in the ≥30-day group. This rapid ART group also featured the smallest share of participants with CD4 cell count ≥500 cells/μL (8.7%), and low baseline viral load (< 1,000 copies/mL) was rare across all three cohorts, ranging from 4.3% to 9.7%. Full stratified CD4 and viral load data are presented in Table 1.

Analysis of marital status showed that more than half of the total cohort were married or cohabiting (50.8%). The rapid ART group had the largest proportion of married/cohabiting participants (53.7%) and the smallest share of single participants (21.1%). Conversely, the ≥30-day group had the highest proportion of single individuals (31.4%). Across the three ART groups, heterosexual transmission accounted for the highest proportion of HIV acquisition, with rates of 82.9%, 77.1%, and 74.6%, respectively; infection via injection drug use was highest in the ≥30-day group (1.2%). Regarding initial ART regimens, NNRTI-based regimens predominated in all three groups, with proportions of 75.0%, 71.3%, and 73.9%, respectively. Notably, the 8–29-day group presented the highest proportion of INSTI-based regimens (21.9% vs. 16.3% and 17.2%) (Table 1).

### 3.2 Trends in rapid ART initiation and time from HIV diagnosis to treatment initiation

**Figure 2:**
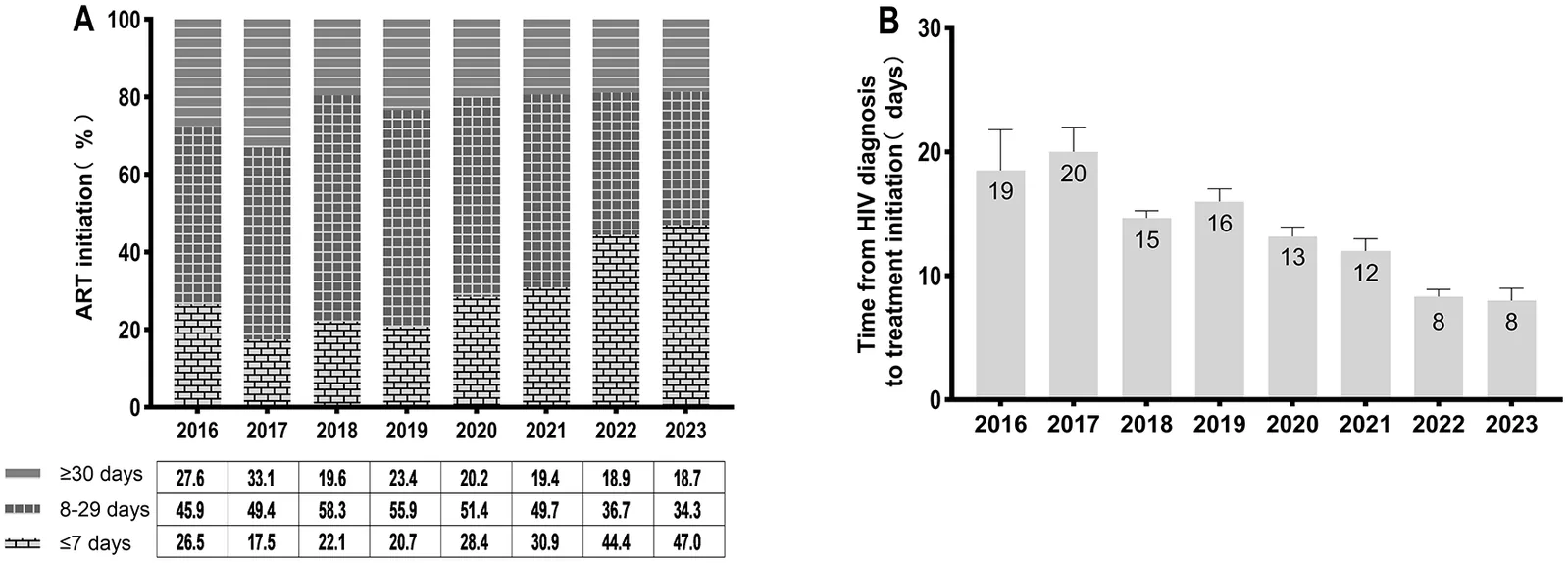
Trends in rapid ART initiation **(A)** and the time from HIV diagnosis to treatment initiation **(B)** from 2016 to 2023. The time interval between diagnosis and treatment is presented as the median with 95% CI. ART, antiretroviral therapy; CI, confidence interval; HIV, human immunodeficiency virus.

As Figure 2A shows, 26.5% of participants in Guizhou Province initiated ART within 7 days of diagnosis in 2016. From 2016 to 2019, the proportion of participants initiating ART within 7 days fluctuated. It increased year by year after 2019 and reached 47.0% in 2023, which was 1.37 times and 2.51 times that of the 8–29-day group and ≥30-day group in the same period, respectively. From 2019 to 2023, the proportion of ART initiation increased annually in the ≤ 7-day group but declined in both the 8–29-day and ≥30-day groups. The annual growth rate of the ≤ 7-day group was approximately 1.2 times the annual decline rate of the 8–29-day group. The median time from diagnosis to ART initiation was 19 days (IQR: 7–32) in 2016, reached a peak of 20 days (IQR: 10–35) in 2017, and then showed a continuous downward trend, stabilizing at 8 days (IQR: 3–22) during 2022–2023 (Figure 2B).

### 3.3 Loss to follow-up

**Figure 3:**
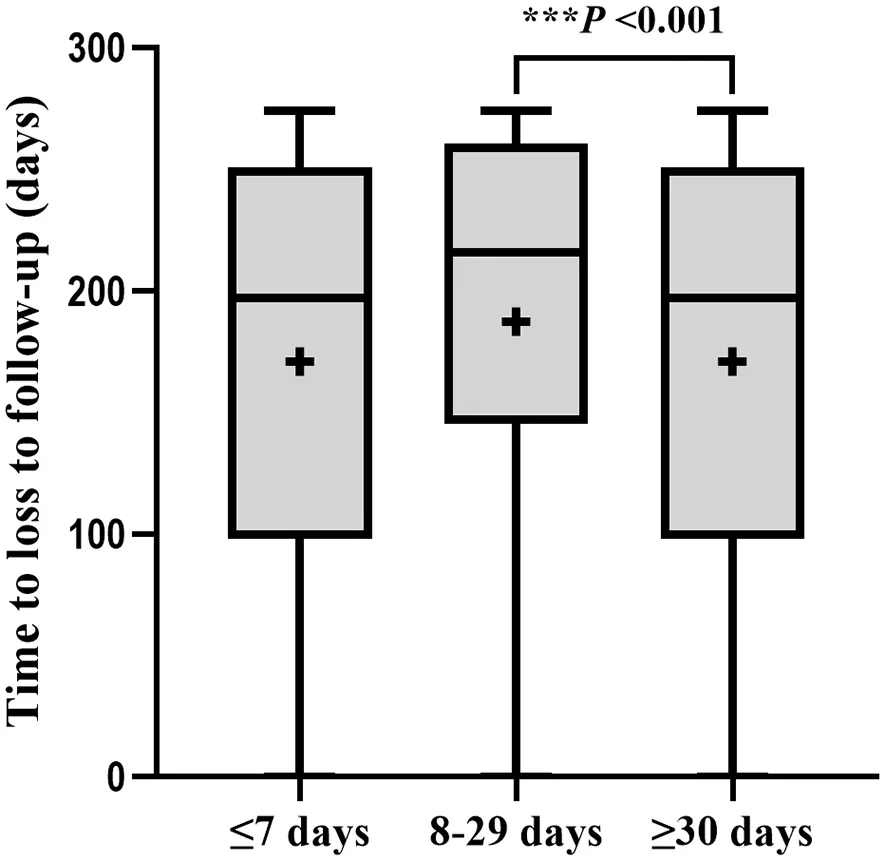
Distribution of time to loss to follow-up stratified by timing of ART initiation among patients with confirmed loss to follow-up.

A total of 8,163 participants contributed 83,989.62 person-months of observation (PMO), with an overall LTFU incidence rate of 2.74 per 100 PMO. When stratified by the time from HIV diagnosis to ART initiation, subgroup LTFU rates were 3.09, 2.29, and 3.28 per 100 PMO for the rapid ART group, 8–29-day group, and ≥30-day group, respectively. A total of 2,299 participants (28.2%) experienced LTFU within 12 months after enrollment, with a median duration to LTFU of 209 days (IQR: 129–257). The highest proportion of 12-month LTFU was reported in 2022 (26.4%). Among rapid ART initiators, 812 (31.2%) experienced LTFU, with a median time to LTFU of 207 days (IQR: 132–254). Delayed ART initiators demonstrated 1,487 LTFU cases, with a median duration to LTFU of 216 days (IQR: 145–261) and 197 days (IQR: 98–251) for the 8–29-day and ≥30-day groups, respectively (Figure 3).

**Figure 4:**
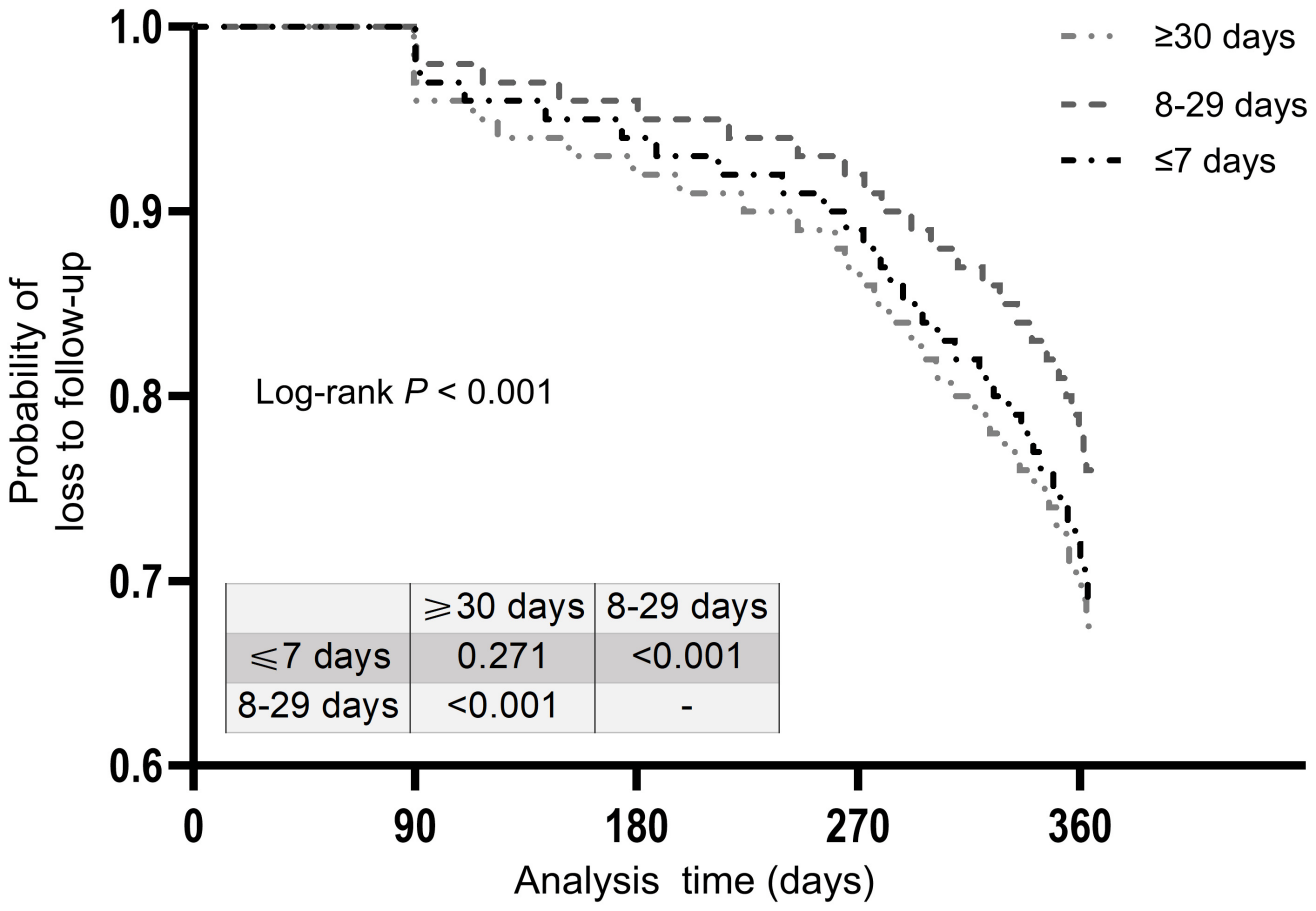
Life table analysis of loss to follow-up stratified by ART initiation postdiagnosis timing ( ≤ 7, 8–29, and ≥30 days). ART: antiretroviral therapy.

By Kaplan–Meier analysis, the LTFU rate among rapid ART initiators differed significantly from that of the 8–29-day group (*P* < 0.001), with no significant difference observed relative to the ≥30-day group (*P* = 0.271, Figure 4). Furthermore, the LTFU rate was significantly lower in the 8–29-day group than in the ≥30-day group (*P* < 0.001). The primary multivariable Cox regression model yielded a Harrell's C-index of 0.588. We constructed an extended time-dependent Cox model to properly account for covariates with violations of the proportional hazards assumption. This final adjusted model demonstrated robust global fit metrics (−2 log-likelihood = 40,448.76; AIC = 40,498.76; BIC = 40,673.95), with complete model performance statistics summarized in Table 2.

**Table 2:** Extended multivariable cox model with time-dependent interactions for loss to follow-up.

Variables	Main effect		Time-dependent Interaction	
	aHR (95% CI)	*P*	aHR (95% CI)	*P*
ART initiation group				
≥30days	Ref		Ref	
8-29days	0.741 (0.662, 0.829)	< 0.001	1.163 (1.035, 1.308)	0.011
≤ 7days	0.911 (0.810, 1.024)	0.12	1.126 (0.999, 1.268)	0.051
Sex				
Female	Ref		Ref	
Male	1.055 (0.955, 1.165)	0.293	0.944 (0.852, 1.046)	0.272
Enrollment period				
2016–2020	Ref		-	
2021–2023	1.366 (1.251, 1.492)	< 0.001		
Age, at Enrollment (years)				
18–29 years	Ref		-	
30–49 years	0.933 (0.804, 1.082)	0.357		
≥50 years	0.946 (0.805, 1.112)	0.502		
Enrollment CD4, (cells/μL)				
< 200	Ref		-	
200–499	1.003 (0.916, 1.097)	0.955		
≥500	0.969 (0.828, 1.135)	0.7		
Baseline viral load, (copies/μL)				
< 1,000	Ref		Ref	
1,000–100,000	0.808 (0.678, 0.962)	0.016	1.126 (0.947, 1.339)	0.179
>100,000	0.773 (0.644, 0.928)	0.006	1.059 (0.887, 1.265)	0.527
Transmission route				
Heterosexual	Ref		Ref	
Homosexual	0.699 (0.597, 0.818)	< 0.001	1.546 (1.274, 1.876)	0.001
Injection drug use	0.900 (0.470, 1.726)	0.752	0.927 (0.494, 1.739)	0.812
Unknown	1.232 (0.985, 1.540)	0.067	0.811 (0.662, 0.993)	0.043
Marital status				
Single	Ref		Ref	
Married/cohabiting	1.032 (0.887, 1.200)	0.685	1.082 (0.944, 1.241)	0.258
Divorced/widowed	1.116 (0.945, 1.319)	0.196	0.986 (0.850, 1.144)	0.856
Model Performance Metrics				
−2 Log likelihood	40,448.76		-	
AIC	40,498.76		-	
BIC	40,673.95		-	
C-index	-		-	

ART, antiretroviral therapy; IQR, interquartile ranges; NNRTI, non-nucleoside reverse transcriptase inhibitor, including EFV, NVP, ETV/N; PI, protease inhibitor, including LPV/r, ATV/r, DRV/r; INSTI, integrase inhibitor, including DTG, RAL, EVG/c, BIC; Other: all initial ART regimens not classified as NNRTI, PI, or INSTI.

Schoenfeld residual tests detected departures from the proportional hazards assumption for multiple covariates (Supplementary Table S2). Visual inspection of log-log survival curves further verified non-proportionality of hazards for our primary exposure, timing of ART initiation (Supplementary Figure S1). The extended multivariable Cox model showed a substantially lower LTFU hazard among participants starting ART within 8–29 days (aHR = 0.741, 95% CI: 0.662–0.829; *P* < 0.001; 8–29 days vs. ≥30 days), controlling for age, sex, enrollment CD4 count, baseline viral load, transmission route, and marital status (Table 2). The hazard of LTFU was greater among participants enrolled during 2021–2023 (aHR = 1.366, 95% CI: 1.251–1.492; *P* < 0.001; 2021–2023 vs. 2016–2020). Conversely, the hazard of LTFU was significantly lower in participants with baseline viral loads of 1,000–100,000 copies/mL (aHR = 0.808, 95% CI: 0.678–0.962; *P* = 0.016) and >100,000 copies/mL (aHR = 0.773, 95% CI: 0.644–0.928; *P* = 0.006; both vs. < 1,000 copies/mL). In addition, homosexual transmission was associated with a reduced LTFU hazard (aHR = 0.699, 95% CI: 0.597–0.818; *P* < 0.001; homosexual vs. heterosexual transmission).

Three covariates exhibited statistically significant time-dependent interaction terms. The interaction estimates for the 8–29-day ART interval (aHR = 1.163, 95% CI: 1.035–1.308; *P* = 0.011) and homosexual transmission (aHR = 1.546, 95% CI: 1.274–1.876; *P* < 0.001) revealed that their baseline protective effects against LTFU weakened gradually with longer follow-up. Among participants with unknown transmission routes, the baseline hazard of LTFU was marginally higher relative to the reference group (aHR = 1.232, 95% CI: 0.985–1.540; *P* = 0.067), whereas the significant time-by-covariate interaction (aHR = 0.811, 95% CI: 0.662–0.993; *P* = 0.043) suggested this relative excess hazard faded progressively over observation time.

Sensitivity analyses confirmed robust findings across all four Cox models. Core predictors, including enrollment between 2021 and 2023, baseline viral load, ART initiation within 8–29 days of enrollment, and heterosexual transmission, maintained consistent effect directions and statistical significance regardless of covariate adjustment and time-varying interaction specifications.

Two variables showed prominent differences across model frameworks: ≤ 7-day ART initiation and unknown transmission route. Static Cox models (Models 1 and 2), which ignored violations of the proportional hazards assumption, identified apparent constant main effects for these two factors. After incorporating time-by-covariate interaction terms in Models 3 and 4, the main effects of the two variables lost statistical significance, and their time-dependent hazard associations were exclusively captured by the interaction terms. These results reveal a major limitation of conventional Cox regression: it can generate biased static effect estimates when the proportional hazards assumption fails to hold.

Furthermore, the “other” subgroup of initial ART regimen independently predicted a lower risk of LTFU, yet the association between rapid ART initiation and LTFU remained highly robust in sensitivity models that additionally adjusted for this regimen (Supplementary Table S3).

### 3.4 Virologic failure

Among 4,265 enrolled participants, a total of 330 individuals were identified with virologic failure after excluding LTFU cases. The virologic failure rates were 7.7% overall. The ≥30-day group had the highest rate at 9.3% (78/839), while the rates were 7.1% (93/1,308) and 7.5% (159/2,118) in the ≤ 7-day group and 8–29-day group, respectively (*P* = 0.154, Figure 1).

We addressed missing laboratory follow-up measurements using MICE, creating 30 distinct complete imputed datasets. All regression-derived model performance statistics were pooled across datasets in accordance with Rubin's rules. The fully adjusted multivariable logistic regression model exhibited acceptable discriminative performance, with a pooled area under the receiver operating characteristic curve (AUC) of 0.651(95% CI: 0.636–0.670). Model calibration was satisfactory overall; the Hosmer–Lemeshow goodness-of-fit test confirmed adequate calibration in 28 out of the 30 imputed datasets.

Multivariable logistic regression identified independent predictors of virologic failure, with rapid ART initiation conferring higher odds of failure compared with delayed ART initiation (aOR = 1.406; 95% CI: 1.020–1.940; *P* = 0.038; ≤ 7 days vs. ≥30 days) (Table 3). Greater baseline CD4+ T cell counts (200–499 cells/μL vs. < 200 cells/μL) were also positively associated with elevated odds of virologic failure (aOR = 1.496; 95% CI: 1.152–1.941; *P* = 0.003). In contrast, two factors were associated with a lower likelihood of virologic failure: male sex (aOR = 0.681; 95% CI: 0.509–0.912; *P* = 0.010; male vs. female), and study enrollment during 2021–2023 relative to the 2016–2020 period (aOR = 0.742; 95% CI: 0.584–0.943; *P* = 0.015; 2021–2023 vs. 2016–2020).

**Table 3:** Multivariable logistic regression analysis of virologic failure using multiple imputation.

Variables	Univariate		Multivariate	
	OR (95% CI)	*P*	aOR (95% CI)	*P*
ART initiation group				
≥30days	Ref		Ref	
8–29days	1,214 (0.918, 1.606)	0.173	1.229 (0.925,1.634)	0.155
≤ 7days	1.317 (0.965, 1.798)	0.082	1.406 (1.020,1.940)	0.038
15.6-7.4,14498ptSex				
Female	Ref		Ref	
Male	0.746 (0.566, 0.983)	0.038	0.681 (0.509,0.912)	0.010
15.6-7.4,14498ptEnrollment period				
2016–2020	Ref		Ref	
2021–2023	0.704 (0.565, 0.876)	0.002	0.742 (0.584,0.943)	0.015
Age, at enrollment (years)				
18–29 years	Ref		Ref	
30–49 years	0.787 (0.540, 1.147)	0.212	1.128 (0.716, 1.778)	0.601
≥50 years	0.561 (0.397, 0.792)	0.001	0.914 (0.556, 1.502)	0.722
Enrollment CD4, (cells/μL)				
< 200	Ref		Ref	
200–499	1.770 (1.390, 2.273)	< 0.001	1.496 (1.152, 1.941)	0.003
≥500	2.047 (1.259, 3.326)	0.004	1.480 (0.891, 2.458)	0.129
Baseline viral load, (copies/μL)				
< 1,000	Ref		Ref	
1,000–100,000	0.936 (0.510, 1.717)	0.831	0.953 (0.513, 1.770)	0.878
>100,000	0.504 (0.275, 0.924)	0.027	0.612 (0.327, 1.147)	0.125
Transmission route				
Heterosexual	Ref		Ref	
Homosexual	1.836 (1.295, 2.601)	< 0.001	1.428 (0.935, 2.181)	0.099
Injection drug use	1.526 (0.187, 12.482)	0.692	1.669 (0.200, 13.913)	0.635
Unknown	1.964 (0.756, 5.099)	0.164	1.944 (0.747, 5.063)	0.172
Marital status				
Single	Ref		Ref	
Married/cohabiting	0.732 (0.532, 1.007)	0.056	0.872 (0.549, 1.384)	0.558
Divorced/widowed	0.517 (0.369, 0.723)	< 0.001	0.656 (0.400, 1.077)	0.095
Model Performance Metrics				
AUC (95% CI)	-		0.651(0.636, 0.670)	
*P*	-		< 0.001	
Hosmer-Lemeshow test (*P*)	-		28/30 imputed datasets *P* > 0.05	

ART, antiretroviral therapy; IQR, interquartile ranges; NNRTI, non-nucleoside reverse transcriptase inhibitor, including EFV, NVP, ETV/N; PI, protease inhibitor, including LPV/r, ATV/r, DRV/r; INSTI, integrase inhibitor, including DTG, RAL, EVG/c, BIC; Other: all initial ART regimens not classified as NNRTI, PI, or INSTI.

Sensitivity analyses restricted to virologic failure confirmed robust associations for several key predictors across all analytical frameworks (Supplementary Table S4). Across all models, baseline CD4^+^ T-cell counts of 200–499 cells/μL independently predicted higher odds of virologic failure. Except for Models 5 and 6, male sex and study enrollment during 2021–2023 were consistently associated with lower odds of virologic failure in all remaining models.

However, statistical significance for the remaining covariates varied by model specification. Notably, multiple imputation models (Models 7 and 8) showed that baseline CD4^+^ T-cell counts ≥ 500 cells/μL were associated with higher odds of virologic failure, whereas a baseline viral load > 100,000 copies/mL correlated with lower odds. Additional protective factors against virologic failure included homosexual transmission (significant in Models 1, 2, 7, and 8) and divorced or widowed marital status (significant in Models 1, 2, and 6). By contrast, INSTI-based regimens were associated with higher odds of virologic failure only in Models 2 and 6 (Supplementary Table S4).

### 3.5 Immunologic failure

Among the 5,025 participants, after excluding those with LTFU,887 were identified with immunologic failure. The total rate of immunologic failure was 17.7%. About 15.4% (232/1,502) of participants in the rapid ART group experienced immunologic failure, with rates of 17.9% (452/2,350) and 20.4% (203/993) in the 8–29-day and ≥30-day delayed ART groups, respectively (*P* = 0.005, Figure 1).

We similarly addressed missing laboratory measurements for the immunologic failure model using MICE, generating 30 distinct complete imputed datasets. The fully adjusted multivariable logistic regression model showed moderate-to-good discriminative ability, with a pooled AUC of 0.744 (95% CI: 0.726–0.761). Overall model calibration was satisfactory; the Hosmer-Lemeshow goodness-of-fit test demonstrated excellent model calibration uniformly across all imputed datasets (all *P* > 0.05).

Logistic regression analysis revealed that participants with rapid ART initiation had higher odds of immunologic failure relative to those with delayed ART initiation (aOR = 1.540; 95% CI: 1.221–1.941; *P* < 0.001; ≤ 7 days vs. ≥30 days) (Table 4). After adjustment for confounding factors, participants with a higher CD4 count had a lower risk of immunologic failure (aOR = 0.309; 95% CI: 0.252–0.378; *P* < 0.001; 200–499 vs. < 200; aOR = 0.065; 95% CI: 0.050–0.086; *P* < 0.001; ≥500 vs. < 200). Participants aged ≥30 years at enrollment had a higher risk of immunologic failure (aOR = 0.643; 95% CI: 0.491–0.842; *P* < 0.001; 30–49 years vs. 18–29 years; aOR = 0.390; 95% CI: 0.287–0.530; *P* < 0.001; ≥50 vs. 18–29). Lower risk of immunologic failure was revealed among males (aOR = 0.707; 95% CI: 0.579–0.863; *P* < 0.001; male vs. female). Higher odds of immunologic failure were observed among participants with a baseline viral load >100,000 copies/mL (aOR = 1.652; 95% CI: 1.184–2.350; *P* = 0.003; >100,000 copies/mL vs. < 1,000 copies/mL).

**Table 4:** Multivariable logistic regression analysis of immunologic non-response using multiple imputation.

Variables	Univariate		Multivariate	
	OR (95% CI)	*P*	aOR (95% CI)	*P*
ART initiation group				
≥30days	Ref		Ref	
8-29days	1.182 (0.987, 1.415)	0.070	1.222 (1.003, 1.489)	0.047
≤ 7days	1.329 (1.081, 1.633)	0.007	1.540 (1.221, 1.941)	< 0.001
Sex				
Female	Ref		Ref	
Male	0.875 (0.741, 1.033)	0.115	0.707 (0.579, 0.863)	< 0.001
Enrollment period				
2016–2020	Ref		Ref	
2021–2023	0.923 (0.802, 1.062)	0.263	0.924 (0.787, 1.086)	0.338
Age, at Enrollment (years)				
18–29 years	Ref		Ref	
30–49 years	1.045 (0.855, 1.278)	0.665	0.643 (0.491, 0.842)	< 0.001
≥50 years	0.713 (0.589, 0.863)	< 0.001	0.390 (0.287, 0.530)	< 0.001
Enrollment CD4, (cells/μL)				
< 200	Ref		Ref	
200–499	0.312 (0.258, 0.378)	< 0.001	0.309 (0.252, 0.378)	< 0.001
≥500	0.074 (0.057, 0.095)	< 0.001	0.065 (0.050, 0.086)	< 0.001
Baseline viral load, (copies/μL)				
< 1,000	Ref		Ref	
1,000–100,000	1.442 (1.090, 1.908)	0.010	1.273 (0.934, 1.735)	0.127
>100,000	2.709 (2.018, 3.637)	< 0.001	1.652 (1.184, 2.305)	0.003
Transmission route				
Heterosexual	Ref		Ref	
Homosexual	1.066 (0.892, 1.273)	0.484	1.179 (0.916, 1.520)	0.201
Injection drug use	3.392 (0.481, 23.935)	0.220	4.169 (0.501, 34.676)	0.186
Unknown	0.889 (0.579, 1.365)	0.590	0.812 (0.504, 1.308)	0.391
Marital status				
Single	Ref		Ref	
Married/cohabiting	0.973 (0.819, 1.156)	0.755	1.251 (0.941, 1.663)	0.123
Divorced/widowed	0.838 (0.684, 1.025)	0.085	1.083 (0.794, 1.477)	0.615
Model Performance Metrics				
AUC (95% CI)	-		0.744 (0.726, 0.761)	
*P*	-		< 0.001	
Hosmer-Lemeshow test (P)	-		All imputed datasets *P* > 0.05	

ART, antiretroviral therapy; IQR, interquartile ranges; NNRTI, non-nucleoside reverse transcriptase inhibitor, including EFV, NVP, ETV/N; PI, protease inhibitor, including LPV/r, ATV/r, DRV/r; INSTI, integrase inhibitor, including DTG, RAL, EVG/c, BIC; Other: all initial ART regimens not classified as NNRTI, PI, or INSTI.

Sensitivity analyses focusing on immunologic failure demonstrated remarkable robustness for several key predictors across all analytical frameworks (Supplementary Table S4). Consistently across all models, male sex, older age groups, and higher baseline CD4^+^ T-cell counts were associated with reduced odds of immunologic failure. In contrast, rapid ART initiation within 7 days and baseline viral load >100,000 copies/mL independently predicted elevated odds of immunologic failure in all models. Participants who initiated ART between 8 and 29 days also had higher odds of immunologic failure in Models 1–6, yet this association lost statistical significance in the multiple imputation models (Models 7–8).

Nevertheless, the statistical significance of multiple covariates differed across model specifications, with the greatest variability seen in Models 3 and 4. Intermediate baseline viral load (1,000–100,000 copies/mL) and homosexual HIV transmission predicted higher odds of immunologic failure exclusively in these two models, whereas an unknown transmission route and enrollment from 2021 to 2023 were linked to reduced immunologic failure odds. INSTI-based regimens exerted protective effects only in Model 4, with no such association identified in other adjusted models. Importantly, although the effect sizes of secondary covariates fluctuated widely, additional adjustment for the initial ART regimen in sensitivity analyses did not shift our primary conclusion: rapid ART initiation remained an independent predictor of elevated immunologic failure risk, with highly consistent effect estimates (Supplementary Table S4).

## 4 Discussion

This large-scale retrospective real-world study explored the association between rapid ART initiation and therapeutic outcomes in Guizhou Province, China from 2016 to 2023. This research indicated that following the implementation of the Treat-All policy, there was a significant and sustained decline in time to ART initiation in Guizhou Province, China. Between 2016 and 2023, the median duration from HIV diagnosis to ART initiation decreased from 19 to 8 days and remained stable. Our study detected no significant difference in LTFU risk between patients with rapid vs. delayed ART initiation. For clinical efficacy endpoints, multivariable logistic regression identified rapid ART initiation as an independent predictor of higher odds of both virologic and immunologic failure. Critically, these adverse associations were not materially changed across alternative model specifications and remained highly robust in all sensitivity analyses.

Following the implementation of the Treat-All policy, 68.1% of PLWH in this cohort initiated ART >7 days after diagnosis, while 20.9% initiated ART >30 days after diagnosis. Furthermore, from 2016 to 2023, the proportion of PLWH initiating ART within 7 days after HIV diagnosis increased to 47.0%.This finding is consistent with previous reports, indicating that the Treat-All policy has significantly improved ART coverage in Guizhou Province (26–28). However, Our study showed that PLWH with high CD4 counts (≥500 cells/μL) were less likely to initiate rapid ART, which was consistent with the findings of some previous studies (29). Meanwhile, our study found that women had overall higher CD4 counts at ART initiation than men, which is consistent with the general consensus that women are more likely to receive early HIV screening and maintain higher medical adherence (30, 31). Despite the relaxation of ART initiation criteria, a large number of PLWH still initiated ART only when their disease progressed to the advanced stage in clinical practice. This phenomenon was closely associated with insufficient testing coverage, poor linkage between diagnosis and treatment, low treatment initiative among PLWH, and regional disparities in medical resources (32–34).

With the continuous expansion of coverage under China's NFATP, the accessibility of ART services in China has steadily increased; however, disjointed diagnosis-treatment service linkage still persists (35). In China, HIV testing and diagnosis are independently undertaken by the CDC, while clinical treatment is provided by medical institutions, leading to the separation of medical care and disease prevention services. The conventional workflow of ART care is cumbersome, requiring repeated outpatient visits and separate laboratory tests, which results in a prolonged overall waiting period for patients. Accordingly, relevant studies have proposed promoting the integrated one-stop care model (36). This integrated one-stop care model has achieved promising practical outcomes in Wuxi (37), However, significant regional disparities in HIV epidemic burden, economic development, and healthcare service capacity are evident across China. Such regional imbalance not only impedes the nationwide rollout and unified implementation of the rapid ART policy, but also leads to marked geographical heterogeneity in the outcomes of integrated diagnosis and treatment service systems. In view of the practical outcomes of integrated care in Guizhou Province, substantial room remains for improving the quality of regional care linkage.

Several observational studies conducted in South Africa suggested that rapid ART initiation might reduce retention in care among PLWH (38, 39). However, this study found no statistically significant difference in the risk of LTFU between the immediate and deferred ART groups at the 12-month follow-up. Furthermore, several observational studies demonstrated that rapid ART initiation could be accompanied by suboptimal long-term retention in care. This phenomenon stemmed from multiple factors at both patient and healthcare system levels, including socioeconomic status, public perception of healthcare professionals' competence, service capacity and quality of counseling interventions in different medical institutions, as well as the accessibility of supportive health services (40, 41). Based on the findings of this study, providing intensive, individualized health counseling for ART-naive PLWH is critical to improving long-term retention in care.

In this study, the crude LTFU rate was higher among patients initiating ART within 7 days versus those starting treatment at 8–29 days (31% vs. 23%). This disparity was mainly attributable to a marked imbalance in enrollment calendar years between the two groups. Specifically, 65.8% of participants in the rapid ART group were enrolled from 2021 to 2023, compared with only 46.2% in the 8–29-day group. Widespread COVID-19-related service disruptions during this period elevated the population-wide baseline hazards of LTFU (42, 43). As such, overrepresentation of the pandemic era within the rapid ART group confounded and inflated its crude LTFU rate. After adjustment for enrollment period and additional confounders, rapid ART initiation exerted no independent effect on LTFU risk, verifying that the unadjusted between-group difference did not stem from the rapid treatment strategy *per se*.

Nevertheless, time-dependent Cox analyses demonstrated that LTFU hazards rose steadily with extended follow-up in both treatment groups, indicating that any protective benefit of early ART initiation is time-limited. Patients who commence lifelong ART hastily without adequate psychological preparation or thorough pre-treatment counseling are prone to developing late adherence fatigue, which eventually culminates in disengagement from care (44). Accordingly, rapid ART initiation should not be simplified to only accelerating medication dispensation; instead, it requires continuous supportive interventions to convert early care linkage gains into durable long-term clinical outcomes.

Our study confirmed that ART initiation ( ≤ 7 days) was independently associated with higher odds of virologic failure. Sensitivity analyses across all eight analytical models further substantiated this core conclusion, showing consistent effect directions and verifying the robustness of this association.

Several potential mechanisms may underlie this adverse association. First, highly compressed pre-treatment windows often lead to insufficient patient preparation and abbreviated adherence counseling, both essential to help patients understand treatment requirements and establish consistent daily pill-taking habits (45). Second, patients commonly experience severe psychosocial distress immediately after HIV diagnosis—including acute anxiety, stigma-related psychological distress, and challenges adapting to chronic disease status—which further impairs consistent adherence to daily ART regimens (46, 47). Collectively, these factors raise the likelihood of early missed doses and suboptimal adherence, ultimately increasing odds of virologic failure. In real-world clinical settings where comprehensive ancillary support services (e.g., adherence counseling, psychosocial interventions) remain under continuous refinement, prematurely shortening pre-treatment preparation may not deliver the anticipated virologic benefits and instead elevate the odds of virologic failure.

Notably, a baseline CD4^+^ T-cell count of 200–499 cells/μL was also identified as an independent predictor of higher odds of virologic failure. Conventional clinical consensus holds that better baseline immune function predicts favorable prognosis, making this counter-intuitive observation explainable via the ‘healthy patient effect'. Patients with preserved baseline immunity tend to underestimate illness severity, which reduces their sense of treatment urgency and drives non-adherent behaviors such as missed doses and irregular dosing (48) Conversely, male sex and study enrollment during 2021–2023 emerged as independent protective factors against virologic failure. The protective effect of male sex may arise from gender-based differences in adherence patterns within this cohort. Meanwhile, the protective effect of later enrollment (2021–2023) likely reflects progressive maturation of local HIV care programs: sustained optimization of ART initiation protocols, expansion of integrated care services, and refined patient education and support frameworks collectively improve patient treatment outcomes.

Suboptimal standardized viral load testing coverage at key post-treatment follow-up time points among PLWH in China partially impedes accurate evaluation of the long-term virologic benefits of rapid ART (49). Therefore, scaling up routine viral load monitoring and refining closed-loop follow-up systems are critical to generating robust real-world clinical evidence, as frequent viral load surveillance has proven clinically valuable in Chinese population-based cohorts (50).

A pivotal finding of our study is that rapid ART initiation exhibited a robust, independent association with elevated odds of immunologic failure at 12 months. This association remained remarkably consistent across all eight sensitivity models and was unaffected by varying missing-data handling strategies and covariate adjustment schemes. Participants initiating ART between 8 and 29 days also presented higher odds of immunologic failure in most models; however, this association lost statistical significance in Model 8, suggesting a potential threshold effect of ART initiation timing. Although this observation contradicts the conventional clinical assumption that earlier ART initiation invariably drives superior immune reconstitution, it aligns closely with emerging evidence from real-world cohort studies (51).

Several underlying mechanisms may explain this counterintuitive association. First, routine clinical care is prone to channeling bias and unmeasured residual confounding: clinicians tend to prioritize rapid ART initiation among patients with severe presenting symptoms, advanced immunosuppression, or rapid disease progression (50). Despite rigorous adjustment for baseline CD4^+^ T-cell count and plasma viral load within our multivariable models, unrecorded indicators of disease severity may still introduce residual confounding. Second, the biological kinetics of CD4^+^ T-cell recovery differ fundamentally from viral decay. While virologic suppression depends largely on antiretroviral regimen potency and treatment adherence, immune reconstitution is largely governed by thymic output capacity, the extent of irreversible lymphoid tissue fibrosis, and pre-treatment chronic immune activation levels. Patients triaged to rapid ART pathways frequently sustain severe, irreversible damage to their immune microenvironment, such that adequate CD4 recovery cannot be attained by 12 months even under sustained complete virologic suppression (52).

Third, the definition of immunologic failure adopted in this study followed standardized clinical criteria (25). Under this definition, patients receiving rapid ART initiation with well-preserved baseline CD4^+^ T-cell counts frequently reached an immune plateau, generating an immune reconstitution “ceiling effect” that limits further CD4 recovery (53). In addition, inherent physiological fluctuations in CD4 counts combined with laboratory measurement error may lead to mild declines in follow-up absolute CD4 values, causing these measurements to drop below elevated baseline levels (54). Consequently, such participants may be categorized as experiencing technical immunologic failure only because they satisfy the threshold of “CD4 count falling to or below baseline,” without true clinical deterioration or progressive immunodeficiency (55). This technical misclassification among individuals with strong baseline immune function partially explains the consistent association between rapid ART initiation and elevated odds of immunologic failure observed across all analytical models.

Although our analysis revealed comparable LTFU risks between patients receiving rapid vs. deferred ART, rapid ART initiation independently increased the odds of subsequent virologic and immunologic failure. This critical trade-off calls for a bifurcated strategy distinguishing population-wide HIV prevention priorities from individualized clinical care.

The paradoxical dissociation identified in this study bears vital clinical implications. The primary limitation of immediate ART rollout is not LTFU or disengagement from care, but a failure to maintain consistent high-standard care across the full treatment cascade even when patients are retained in care. Such structural inequities are pronounced in Guizhou, a mountainous province with constrained economic resources. Heavy travel costs and associated lost work income hinder attendance at frequent early follow-up visits, increasing risks of missed clinic appointments and drug stock interruptions (36, 56). Among ethnic minority populations, language gaps, low community health literacy, and pervasive HIV stigma further undermine abbreviated pre-ART counseling packages designed for rapid ART delivery (57). Cumulative structural barriers explain why the well-documented survival and prognostic benefits of immediate ART observed globally fail to replicate in our regional cohort (51, 58). Insufficient pre-treatment health education paired with a scarcity of sustained adherence interventions acts synergistically to raise concurrent risks of virologic suppression failure and suboptimal immune reconstitution (59, 60).

From a population-level public health standpoint, rapid ART initiation remains a core intervention priority, as it uniquely interrupts HIV transmission chains, accelerates progress toward the UNAIDS 95-95-95 targets, and expands overall treatment coverage (61, 62). Conversely, frontline clinicians should recognize that immune reconstitution exhibits marked interindividual heterogeneity shaped by patients' biological and psychosocial attributes (63). For high-risk individuals—particularly those with elevated baseline viral loads or profound immunosuppression—rigorous long-term immunologic monitoring and tailored adjunctive interventions are necessary to sustain durable immune recovery, even among patients who received rapid ART promptly (64).

Furthermore, to evaluate whether initial ART regimen functions as a mediator and thus induces overadjustment bias, we built paired models—one adjusted for initial regimen and the other unadjusted—across all sensitivity analyses. The highly consistent effect estimates derived from these paired models across every clinical endpoint indicate that initial treatment regimen does not lie on a key mediating pathway along the causal chain. Accordingly, overadjustment bias exerted minimal influence on our primary study conclusions.

Notably, given the lengthy study window spanning 2016–2023, the calendar year of ART initiation may simultaneously modify both treatment timing and long-term clinical outcomes. Although the enrollment period was incorporated a priori as an adjusted covariate in all Cox and logistic regression models to address this temporal confounding, this time-based variable exerted distinctly different effects across study endpoints:

First, for the LTFU outcome, participants enrolled between 2021 and 2023 consistently showed significantly higher hazards of attrition across all Cox model specifications. This suggests that despite access to optimized modern antiretroviral regimens, patients starting therapy in later years faced elevated risks of disengagement from care. This counterintuitive trend is largely explained by restricted healthcare access during the COVID-19 pandemic (43), coupled with potential deficits in health literacy or insufficient treatment readiness among individuals recruited during nationwide rapid ART scale-up (60).

Second, temporal associations of virologic and immunologic failure were highly consistent across all model specifications and missing data handling strategies. This striking divergence further corroborates the robustness of our virologic and immunologic outcomes, while delineating divergent statistical resilience among distinct clinical endpoints in heterogeneous real-world clinical cohorts.

This study has several limitations. First, our study is prone to notable selection bias stemming from strict eligibility criteria. Among the original cohort of 57,892 patients, only 8,163 participants (14.1%) with complete baseline laboratory data and sufficient follow-up were retained for analysis. Relative to excluded individuals, the analytical sample was younger, contained higher proportions of male patients and men who have sex with men, and had a 4.5-fold higher uptake of INSTI-containing regimens. Importantly, excluded patients presented substantial missing baseline biomarker data, with 40.3% missing CD4 counts and 90.1% missing viral load measurements. This strongly suggests that this excluded subgroup comprises of highly vulnerable patients who experienced early mortality, rapid loss to follow-up, or substantial structural barriers preventing baseline testing.

Such informative censoring creates a selected “survivor” cohort, which likely underestimates real-world overall attrition and overestimates population-level virologic suppression rates. Caution is required when generalizing our results, as our findings primarily apply to patients capable of completing full baseline workups and maintaining continuous care. Ultimately, this methodological constraint represents an inevitable trade-off: to preserve complete longitudinal data required for assessing 12-month virologic and immunologic endpoints, some loss of population representativeness was unavoidable.

Second, the exclusion of participants LTFU—who accounted for approximately 28% of the total cohort—in our primary analyses may introduce attrition bias. Disengagement from clinical care is strongly correlated with poor clinical outcomes, which raises concerns about differential loss to follow-up. However, comprehensive sensitivity analyses revealed that effect estimates for virologic and immunologic failure remained highly stable across all alternative models under distinct missing-data assumptions. Such uniform results demonstrate the strong robustness of our main findings and substantiate that the dual adverse risks linked to rapid ART initiation are highly credible.

Third, multiple covariates were tested across multivariable regression and comprehensive sensitivity analyses, which introduces inherent risks of Type I error secondary to repeated statistical testing. We omitted formal multiplicity correction for two methodological justifications. For one, all analytical plans were prespecified a priori under established epidemiologic causal structures, precluding unplanned post-hoc data mining. For another, stringent corrections including Bonferroni penalize statistical power and frequently obscure associations with clinical importance. Even so, the uniform direction of effect estimates across all sensitivity models—evident for both virologic and immunologic failure risks—markedly diminishes the chance that our observed dual adverse associations arise solely from random noise.

Four, the external validity of our findings is constrained by several key limitations. The study cohort was geographically confined to a specific region, which limits the generalizability of our results to national real-world HIV populations. Furthermore, the longitudinal follow-up period partially overlapped with the COVID-19 pandemic. Extensive pandemic-induced disruptions—including altered healthcare resource allocation, fluctuating long-term medication adherence, and adjusted routine follow-up protocols—may have introduced unmeasured variations in clinical outcome trajectories across the cohort.

Lastly, as a registry-based retrospective cohort study, our database lacks granular records of key concurrent clinical comorbidities, including tuberculosis (TB) co-infection and other opportunistic infections (OIs), alongside longitudinal quantified metrics of ART medication adherence. These clinical factors are well-documented critical confounders that substantially shape both clinical decisions on ART initiation timing and subsequent long-term treatment endpoints. The absence of these covariates inevitably introduces unmeasured residual confounding within our multivariable regression models. Accordingly, our study findings require cautious interpretation: unaccounted heterogeneity in comorbid disease burden and adherence patterns may distort the true magnitude of the observed associations between rapid ART initiation and long-term virologic/immunologic outcomes.

## 5 Conclusion

Taken together, China's national Test-and-Treat policy has markedly shortened the time from HIV diagnosis to ART initiation for newly diagnosed PLWH across Guizhou. Paradoxically, rapid ART initiation independently predicted higher dual risks of virologic and immunologic failure relative to deferred ART; this association persisted consistently in all sensitivity analyses. Our findings indicate that in regions with fragmented care linkage, accelerating ART onset alone fails to sustain favorable long-term treatment outcomes. Optimized rapid ART delivery requires personalized intensive adherence counseling, regular laboratory surveillance, and integrated care frameworks that combine immediate treatment with continuous retention support. Ultimately, narrowing gaps in regional medical infrastructure and scaling up routine viral load testing are critical to unlocking the full prognostic benefits of Test-and-Treat in resource-limited areas and meeting UNAIDS 95-95-95 targets.

## Data Availability

The data analyzed in this study is subject to the following licenses/restrictions: The data supporting the findings of this study are available from the corresponding author upon reasonable request. Requests to access these datasets should be directed to PL, lipeng691@126.com.
